# Should all acutely ill children in primary care be tested with point-of-care CRP: a cluster randomised trial

**DOI:** 10.1186/s12916-016-0679-2

**Published:** 2016-10-06

**Authors:** Jan Y. Verbakel, Marieke B. Lemiengre, Tine De Burghgraeve, An De Sutter, Bert Aertgeerts, Bethany Shinkins, Rafael Perera, David Mant, Ann Van den Bruel, Frank Buntinx

**Affiliations:** 1 Nuffield Department of Primary Care Health Sciences, University of Oxford, Radcliffe Primary Care Building, Woodstock Road, Oxford, OX2 6GG UK; 2 Department of Public Health and Primary Care, KU Leuven, Kapucijnenvoer 33J, 3000 Leuven, Belgium; 3 Department of Family Medicine and Primary Health Care, Ghent University, De Pintelaan 185, Gent, 9000 Belgium; 4 Leeds Institute of Health Sciences, University of Leeds, 101 Clarendon Road, Leeds, LS29LJ UK; 5 Research Institute Caphri, Maastricht University, Universiteitssingel 40, Maastricht, 6229 ER The Netherlands

**Keywords:** Serious infection, Child, C-reactive protein, Point-of-care, Primary care

## Abstract

**Background:**

Point-of-care blood C-reactive protein (CRP) testing has diagnostic value in helping clinicians rule out the possibility of serious infection. We investigated whether it should be offered to all acutely ill children in primary care or restricted to those identified as at risk on clinical assessment.

**Methods:**

Cluster randomised controlled trial involving acutely ill children presenting to 133 general practitioners (GPs) at 78 GP practices in Belgium. Practices were randomised to undertake point-of-care CRP testing in all children (1730 episodes) or restricted to children identified as at clinical risk (1417 episodes). Clinical risk was assessed by a validated clinical decision rule (presence of one of breathlessness, temperature ≥ 40 °C, diarrhoea and age 12–30 months, or clinician concern). The main trial outcome was hospital admission with serious infection within 5 days. No specific guidance was given to GPs on interpreting CRP levels but diagnostic performance is reported at 5, 20, 80 and 200 mg/L.

**Results:**

Restricting CRP testing to those identified as at clinical risk substantially reduced the number of children tested by 79.9 % (95 % CI, 77.8–82.0 %). There was no significant difference between arms in the number of children with serious infection who were referred to hospital immediately (0.16 % vs. 0.14 %, *P* = 0.88). Only one child with a CRP < 5 mg/L had an illness requiring admission (a child with viral gastroenteritis admitted for rehydration). However, of the 80 children referred to hospital to rule out serious infection, 24 (30.7 %, 95 % CI, 19.6–45.6 %) had a CRP < 5 mg/L.

**Conclusions:**

CRP testing should be restricted to children at higher risk after clinical assessment. A CRP < 5 mg/L rules out serious infection and could be used by GPs to avoid unnecessary hospital referrals.

**Trial registration:**

ClinicalTrials.gov Identifier: NCT02024282 (registered on 14^th^ September 2012).

## Background

The care for acutely ill children has traditionally been a primary care responsibility [1], but increasing numbers are being seen in secondary care. There has been a 40 % increase in the number of children presenting to the emergency department over the last decade, 14 % of whom present with febrile illness [2]. Urgent hospital admission rates have increased by 28 % in the same period, mostly for acute infections [3], with 23 per 1000 children admitted annually for a condition that could be managed in the community [3, 4].

In contrast, serious infections have become rare and are now estimated to constitute < 1 % of childhood infections presenting to primary care [5]. Pneumonia represents four-fifths of all cases, followed by urinary tract infections, and now very few cases of sepsis, meningitis, or osteomyelitis [6–8], which, although rare, their prompt recognition is essential to avoid complications or death [9]. This is challenging in primary care because the clinical presentations are highly non-specific, especially in the early stages of illness. Only one clinical decision rule has been developed for primary care, with a sensitivity of 100 % and a specificity of 81 % [8]. Although it can be used to rule out serious infections safely, it also results in approximately 20 % of acutely ill children being classified as at higher risk for a serious infection.

Introducing better diagnostic tests might strengthen the primary care management of acutely ill children. Inflammatory markers such as C-reactive protein (CRP) and procalcitonin can assist in diagnosing serious infections in hospital settings [10]. Up until now, such blood tests play only a marginal role in primary care, because the result comes back from the laboratory too late to influence clinical decision-making [7]. Point-of-care tests that perform a CRP test within 4 minutes have now become available [11, 12].

In this study, we aimed to assess whether performing point-of-care CRP testing should be done in all children presenting with acute infection in primary care or only in those deemed at high-risk of serious illness after initial clinical assessment. We also aimed to investigate how CRP results should be interpreted; specifically, whether a low CRP level can rule out infection and the consequent need for hospital referral.

## Methods

We conducted a cluster randomised controlled trial comparing CRP testing in all children with clinically-guided CRP testing. Practices were randomised to undertake point-of-care CRP testing in all children or only children assessed as being at higher risk by a validated clinical decision rule.

Children aged 1 month to 16 years presenting with an acute illness for a maximum of 5 days were recruited consecutively from February 15, 2013, to February 28, 2014, in 78 general practices across Flanders, involving 133 general practitioners (GPs). Children were excluded if the acute illness was caused by purely traumatic or neurological conditions, intoxication, a psychiatric problem, or an exacerbation of a known chronic condition. When the same child was recruited twice within 5 days, we considered the second registration to be part of the same illness episode and excluded the second registration from the analyses. If a physician recruited fewer than five children over the 1-year study period, consecutive inclusion was assumed to be violated, leading to the exclusion of that physician from the analysis.

In the “CRP for all” group, each child had a CRP test. In the “CRP only if at clinical risk” group, CRP testing was dependent on the presence of at least one of the following clinical features: breathlessness, body temperature of at least 40 °C, diarrhoea in children 12–30 months of age, and clinician concern [6]. “Breathlessness” was defined as difficult or laboured breathing. “Body temperature” was defined as the highest body temperature measured during the illness episode by the parents or the physician according to their usual practice. Before analysis, 0.5 °C was added to temperatures measured under the axilla or with a tympanic thermometer [13, 14]. “Diarrhoea” was defined as loose or watery stools, increased in frequency and volume [15]. “Clinician concern” was defined as a subjective feeling of the physician that something was out of the ordinary.

We used the Afinion™ CRP Test Cartridge, which has a measuring range for CRP of 5–200 mg/L [12] and requires 1.5 μL of blood obtained by finger prick, providing a result within 4 minutes. We trained all physicians to perform the CRP test. Internal quality control was performed according to the manufacturer’s instructions.

Randomisation was performed at the practice level to avoid contamination and stratified by practice type (solo, duo, group) before randomisation. The intervention-specific protocols were briefed during a face-to-face meeting at each practice, differing primarily in the indication to test for CRP.

The primary outcome of the study was hospital admission (> 24 hours) for a serious infection within 5 days after initial presentation. Hospital admission was verified by a search of the electronic medical records of all hospitals in the practices’ catchment area, an interview with each participating GP and a diary completed by parents. Serious infections were defined as:

- sepsis (including bacteraemia): pathogenic bacteria isolated from blood culture
- meningitis: pleocytosis or identification of bacteria or a virus in cerebrospinal fluid
- appendicitis: histology
- pneumonia: infiltrate on chest X-ray
- osteomyelitis: pathogens from bone aspirate or a MRI or bone scan suggestive for osteomyelitis
- cellulitis: acute suppurative inflammation of the subcutaneous tissues
- bacterial gastroenteritis: pathogen isolated from stool culture
- complicated urinary tract infection: > 10^5^/mL pathogens of a single species isolated from urine culture and systemic effects such as fever

In cases where no definitive adjudication could be made based on the above criteria, an adjudication committee, consisting of clinicians with expertise in acute paediatric care, assigned outcome by consensus, using all available information.

Secondary outcomes were referrals (immediate or delayed) to secondary care (hospital-based paediatricians) and the ordering of additional testing (including blood and urine tests, and imaging) at the initial presentation as recorded by the GP.

Sample size calculation was described previously [8], and is based on the assumptions that prevalence would be 0.8 % and sensitivity and specificity of CRP would be 75 %, as found in a recent meta-analysis, using bivariate random effects meta-analysis across a range of CRP cut-off values [10]. Differences in baseline characteristics and clinical features were analysed through χ^2^ testing and nonparametric equality-of-medians testing to assess potential recruitment bias. We calculated accuracy of CRP in both groups for the following pre-determined thresholds: 5 and 200 mg/L which are the lower and upper limit of the point-of-care CRP test, and 20 and 80 mg/L which have been identified as ‘ruling out’ and ‘ruling in’ thresholds, respectively, in secondary care [10]. We examined whether time from onset of fever (in days) influenced the level of CRP using non-parametric equality-of-medians tests [16]. The 4.2 % missing values for CRP in those patients that should have had a CRP test were assigned to either side of the optimal split for this continuous predictor, defined as the split resulting in the smallest *P* value through χ^2^ testing for the difference between both sides of the split [17].

To test whether there were any differences in timely diagnosis, referrals to secondary care or additional testing between the two groups, we conducted a mixed-effects logistic regression analysis to account for the clustering at practice level, and other potential interaction terms, such as the child’s age, using the xtmelogit function in Stata [18]. All analyses were performed with Stata software (version 11.2; Stata Corp., USA), and JMP Statistical Discovery (version Pro 12.1.0; SAS Institute Inc., USA).

## Results

**Fig. 1:**
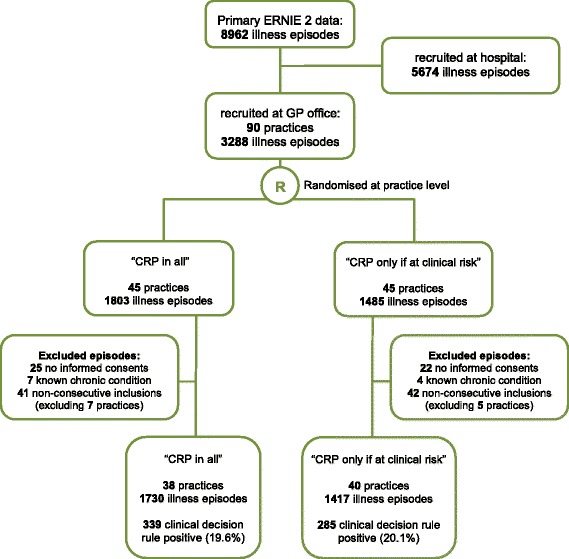
Flowchart of recruited illness episodes in acutely ill children. GP: general practitioner; CRP: point-of-care C-reactive protein testing

The 3147 illness episodes occurred in 2773 children between February 15, 2013, and February 28, 2014 (Fig. 1). The children’s median age was 3.2 years (interquartile range 1.5–6.9) and 1659 were male (52.7 %). Table 1 shows that the randomisation arms were well balanced in terms of sex and the presenting features of the illness, although there was a small imbalance in age (median age 3.6 in the CRP for all arm versus 2.7 years in the CRP if at clinical risk arm).

**Table 1:** Characteristics of children at presentation by randomisation arm

	CRP in all children (*n* = 1730)		CRP only in children at clinical risk (*n* = 1417)		*P* value
	*n*	% or IQR	*n*	% or IQR	
Male	942	53.4 %	735	51.9 %	0.36
Age (median)	3.6	1.6–8	2.7	1.3–5.5	< 0.0001
Fever ≥ 38 °C	961	85.7 %	949	87.6 %	0.19
Duration of fever	2	1–2	2	1–2	0.99
Diarrhoea or vomiting	409	24.2 %	347	25.0 %	0.61
Breathlessness	99	6.0 %	65	4.7 %	0.13
Body temperature ≥ 40 °C	119	7.6 %	120	9.5 %	0.09
Diarrhoea and 12–30 Months	75	4.4 %	77	5.6 %	0.15
Clinician concern	192	11.6 %	148	10.8 %	0.48

*CRP* point-of-care CRP testing, *IQR* interquartile range

### Impact of testing strategy on clinical management

Table 2 shows that restricting CRP testing to children at clinical risk (because of breathlessness, temperature ≥ 40 °C, diarrhoea and age 12–30 months, or clinician concern) substantially reduced the number of children tested to 285/1417 (20.1 %, 95 % CI, 18.0–22.2 %). All children with serious infection had one of the at-risk features sought by the clinical decision rule and thus all received CRP testing. Although fewer children were referred to hospital in the restricted testing arm (2.1 % vs. 2.9 %), the difference lacks statistical significance (*P* = 0.15). Similarly, the reduction in the number of children with serious infection whose admission was delayed (> 24 hours) in the restricted testing arm (0.14 % vs. 0.29 %) was also non-significant.

**Table 2:** Clinical management and main outcome by randomisation arm

	CRP in all children (*n* = 1730)		CRP only in children at clinical risk (*n* = 1417)		Odds ratio adjusted for age and clustering	
	*n*	%	*n*	%	AOR	(95 % CI)
CRP tested	1730	100 %	285	20.1 %	–	
Referred to hospital	50	2.9 %	30	2.1 %	0.61	(0.31–1.21)
Serious infection	7	0.40 %	4	0.28 %	0.61	(0.15–2.39)
Not CRP tested	0	0	0	0	–	
Referred immediately	2	0.16 %	2	0.14 %	1.16	(0.16–8.41)
Delayed admission	5	0.29 %	2	0.14 %	0.43	(0.07–2.53)

*AOR* adjusted odds ratios, *CRP* point-of-care CRP testing, *95 % CI* 95 % confidence intervals

### Diagnostic performance of CRP

Restricting CRP testing to those identified as at clinical risk increased the median CRP level of the children tested from 7 mg/L (IQR: 5–23 mg/L) to 11 mg/L (IQR: 5–30 mg/L). Time from onset of fever did not influence the median point-of-care CRP level (*P* > 0.06).

The impact on diagnostic performance of CRP is shown in Table 3. The small number of cases of serious infection means that the confidence intervals around the number of missed cases are wide, with no indication of a difference between randomisation arms. However, at a CRP threshold of 5 mg/L, no cases of serious infection were missed in either arm. The number of false alarms expressed was also similar in both arms, irrespective of the threshold applied. If the 5 mg/L threshold was adopted as a criterion for hospital referral, over half (59.2 % overall) of the children tested with CRP would be referred. Of these children, 1 in 40 in the restricted testing arm would have serious infection (positive predictive value: 2.4 %). If all children were tested and those with a CRP level ≥ 5 mg/L referred, then the positive predictive value would be even lower (0.8 %) and only 1 in 130 children referred would have a serious infection.

**Table 3:** Diagnostic performance of CRP in diagnosing serious infection at different thresholds by randomisation arm

	CRP in all children			CRP only in children at clinical risk		
Missed cases	n/N	%	95 % CI	n/N	%	95 % CI
≥ 5 mg/L	0/7	0	0.0–40.9	0/4	0	0.0–60.2
≥ 20 mg/L	3/7	42.9	9.9–81.6	3/4	75	19.4–99.4
≥ 80 mg/L	6/7	85.7	42.1–99.6	4/4	100	39.7–100
≥ 200 mg/L	6/7	85.7	42.1–99.6	4/4	100	39.7–100
False alarms	n/N	%	95 % CI	n/N	%	95 % CI
≥ 5 mg/L	918/1723	53.3	49.9–56.8	161/281	57.3	51.3–63.2
≥ 20 mg/L	448/1723	26.0	23.9–28.1	85/281	30.2	24.9–36.0
≥ 80 mg/L	65/1723	3.8	2.9–4.8	14/281	5.0	2.8–8.2
≥ 200 mg/L	2/1723	0.1	0.0–0.4	2/281	0.7	0.0–2.5

*CRP* point-of-care CRP testing, *95 % CI* 95 % confidence intervals

### Children referred to hospital with normal CRP

Twenty-four children were referred to hospital with a low CRP level (< 5 mg/L). The clinical presentation and stated reasons for referral are shown in Table 4. In 17 cases (70.8 %), the referral was made to rule out serious infection. In only one of these cases was the child admitted – a case of viral gastroenteritis where the child was admitted because of dehydration.

**Table 4:** Children referred to hospital with normal CRP level < 5 mg/L (n = 24)

#	Reason for referral to hospital	Additional tests ordered by GP	Highest temperature (°C)	Clinician concern (GP)	Admitted to hospital	Final discharge diagnosis
1	Rule out pneumonia	Chest X-ray; urine culture	40.2	Yes	No	Viral URTI
2	Rule out meningitis	Urine culture	38.2	Yes	No	Viral URTI
3	Rule out meningitis	Urine culture	39.7	Yes	No	Viral URTI
4	Rule out ketoacidosis	Glycaemia fingerstick	35.9	Yes	Yes	Viral URTI
5	Rule out pneumonia	Urine culture	40.5	Yes	No	Viral URTI
6	Rule out UTI	None	40	Yes	No	Viral URTI
7	Might need intensive aerosol treatment	Urine culture	39.6	Yes	No	Viral URTI
8	Rule out pneumonia	FBC; chest X-ray	38.5	Yes	No	Viral URTI
9	Recurring otitis media	Urine culture	37.5	Yes	No	Viral URTI
10	Rule out pneumonia	Urine culture	38	Yes	No	Viral URTI
11	Not resolving 3 days after start antibiotics	Urine culture	38.8	Yes	No	Viral URTI
12	Rule out pneumonia	FBC; ultrasound	–	No	No	Viral URTI
13	Recurring otitis media	none	36.8	No	No	Viral URTI
14	Rule out UTI	Urine culture	38.4	Yes	No	UTI
15	Rule out UTI	Urine culture	40.5	Yes	No	UTI
16	Rule out UTI	Urine culture	36.1	No	No	UTI
17	Rule out UTI	Urine culture	36.6	Yes	No	Viral gastroenteritis
18	Grunting	Chest X-ray; ultrasound	37.1	Yes	No	Viral gastroenteritis
19	Rule out bacterial gastroenteritis	Stool culture	–	Yes	Yes	Viral gastroenteritis
20	Rule out appendicitis	None	37.6	Yes	No	Viral gastroenteritis
21	Rule out appendicitis	None	35.6	No	No	Viral gastroenteritis
22	Rule out appendicitis	Ultrasound; stool culture	–	No	No	Viral gastroenteritis
23	Rule out parasites	FBC; urine culture	37.0	No	No	Viral gastroenteritis
24	Rule out appendicitis	Ultrasound	36.4	No	No	Viral gastroenteritis

*CRP* point-of-care CRP testing, *GP* general practitioner, *URTI* upper respiratory tract infection, *UTI* urinary tract infection, *FBC* full blood count

## Discussion

### Main findings

In primary care, CRP testing can be restricted to children at higher risk of serious infection after clinical assessment. At a threshold of 5 mg/L, CRP still has limited diagnostic value in ruling in serious infection (at most, 1 in 40 children will have serious infection) but it does rule out serious infection and the need for hospital referral.

### Strengths and limitations

This trial was performed in a large number of practices involving 133 general practitioners and 3147 children. The results therefore reflect a real-life implementation of point-of-care CRP testing. Although blinding is not possible in cluster randomised controlled trials, we believe the verification of our target condition is robust and not influenced by the interventions by using a combination of objective criteria and independent adjudicators. We also think that work-up bias in primary care is unlikely to have had a major impact on our assessment of diagnostic performance because the diagnosis of serious infection was based on hospital assessment [19]. We were not able to provide physicians with instructions on how to deal with the CRP result because there were no studies available in primary care to underpin this advice. This could explain the lack of any effect of CRP tests on additional testing or referrals; earlier studies on CRP for antibiotic prescribing in adults have shown that instructions are pivotal for changing physician prescribing behaviour [20]. The trial did not include a no CRP-testing arm and clinical assessment of all children was guided by a validated clinical decision rule. This means that we could not report the effect of CRP testing compared to routine clinical care. It also means that the advantages we report from limiting CRP testing to children at risk will be achieved only if the quality of risk assessment is comparable to that implemented in the trial. Given the simplicity of the decision rule used, this should be achievable in most clinical settings.

### Comparison with existing literature

A systematic review based on studies from hospital settings suggested that CRP levels < 20 mg/L provided the best rule out value for serious infections in children [10]. We applied a lower threshold in a primary care setting because children are presenting at an earlier stage of their illness (i.e. CRP levels in children with evolving serious infection are likely to be lower than when they arrive in hospital) [21].

Most studies on the use of CRP in primary care have focused on antibiotic prescribing in adult patients; a cluster randomised controlled trial has shown that GP’s use of point-of-care testing for CRP significantly reduced antibiotic prescribing if combined with enhanced communication skills [22]. Data from a multinational randomised controlled trial in adults suggested that CRP can be viewed as a tool to decrease diagnostic uncertainty and reassure patients in primary care [23], concluding that addition of CRP at > 30 mg/L improves diagnostic accuracy of a clinical decision rule to predict pneumonia in patients with acute cough [24].

### Implications

Our results support the implementation of clinically guided CRP testing to help rule out serious infection and the need for hospital admission. To detect one child with a serious infection, the clinical decision rule would flag 57 children as potentially having a serious infection. A CRP test in these children allows a serious infection to be excluded in a further 22, which means fewer would have to be referred or receive additional testing. This will strengthen the primary care assessment of acutely ill children, assisting GPs in identifying children with a serious infection without swamping secondary care services. By supporting clinical decision-making, it empowers clinicians to safely manage children in ambulatory care, as prioritized by the NHS Five Year Forward View and the Future Hospital Commission [25, 26]. In clinical practice, the implementation of our findings should always allow for clinical judgment, especially when diagnostic uncertainty remains. In such cases, appropriate safety netting strategies (e.g. re-consultation, telephone follow-up, explicating alarm signs) [27, 28] should be in place. Further research should focus on the implementation of this validated clinical assessment in combination with CRP testing and evaluate the cost-effectiveness of the diagnostic strategy overall.

## Conclusions

CRP testing in primary care should be restricted to children at higher risk after clinical assessment. A CRP < 5 mg/L rules out serious infection and could be used by GPs to avoid unnecessary hospital referrals.

## Abbreviations

CRP: C-reactive protein
GP: General practitioner
IQR: Interquartile range
