# Erratum to: Should all acutely ill children in primary care be tested with point-of-care CRP: a cluster randomised trial

**DOI:** 10.1186/s12916-017-0861-1

**Published:** 2017-05-02

**Authors:** Jan Y. Verbakel, Marieke B. Lemiengre, Tine De Burghgraeve, An De Sutter, Bert Aertgeerts, Bethany Shinkins, Rafael Perera, David Mant, Ann Van den Bruel, Frank Buntinx

**Affiliations:** 1 Nuffield Department of Primary Care Health Sciences, University of Oxford, Radcliffe Primary Care Building, Woodstock Road, Oxford, OX2 6GG UK; 2 Department of Public Health and Primary Care, KU Leuven, Kapucijnenvoer 33 J, 3000 Leuven, Belgium; 3 Department of Family Medicine and Primary Health Care, Ghent University, De Pintelaan 185, 9000 Gent, Belgium; 4 Leeds Institute of Health Sciences, University of Leeds, 101 Clarendon Road, LS29LJ Leeds, UK; 5 Research Institute Caphri, Maastricht University, Universiteitssingel 40, 6229 ER Maastricht, The Netherlands

## Erratum

After publication of the original article [1], it was brought to the authors attention that the discussion section needed an extension regarding issues raised during the peer review process.

As stated in the discussion section of the paper, we acknowledge that the lack of a usual care arm in the trial is unfortunate, and we believe that we have limited the conclusions accordingly. Moreover, because CRP results were disclosed immediately (so that they could influence decision-making which was the purpose of the trial), it is impossible to make any assumptions on what usual care without CRP would have been.

We acknowledge that CRP should not be used routinely in primary care. Our trial shows that restricting CRP testing to at risk children based on their clinical presentation increases CRP’s diagnostic accuracy. Whether referral rates would change compared to usual care remains to be seen; in the context of missed diagnoses (only 4 of 11 seriously ill children were referred immediately in our trial, Table 2 of the original paper) [1], we would expect CRP to increase referral accuracy rather than decrease total referral rates.

Serious infections in children are very rare in primary care [2, 3], which is one of the reasons why they are difficult to diagnose. In an ideal world, we would like to achieve perfect sensitivity and specificity. But missed diagnoses of serious infections are potentially very dangerous whereas unnecessarily referring a child for secondary care assessment is annoying and costly but not directly impacting on the child’s prognosis. For that reason, we believe that in primary care, ruling out serious infections should be prioritised over ruling in.

The clinical prediction rule was designed to rule out serious infections in as many children as possible, but resulted in approximately 20% of children classified as ‘at risk’. The purpose of adding CRP to the diagnostic assessment was to further rule out serious infections in those 20%. Adding CRP to the clinical prediction rule increases specificity from 80% to 89% while keeping sensitivity at 100%, which is shown in Fig. 1.

**Fig. 1:**
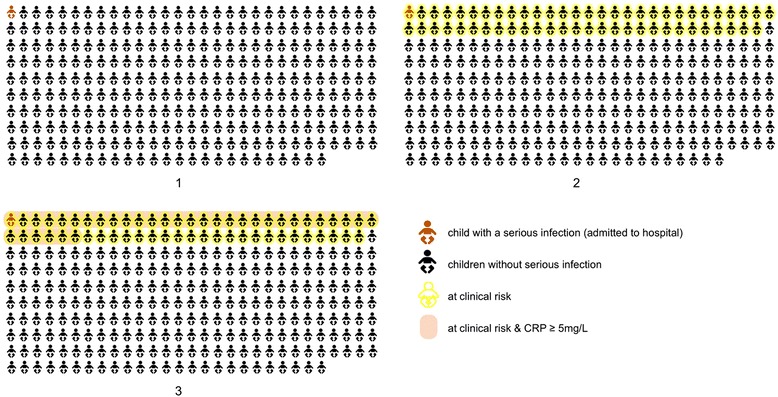
To detect one child with a serious infection, the clinical decision rule would flag 57 children as potentially having a serious infection. A CRP test in these children allows a serious infection to be excluded in a further 22, which means fewer would have to be referred or receive additional testing.

Considering the restricted CRP strategy results in fewer CRP tests while maintaining perfect sensitivity and improving specificity, it is highly likely this will be cost-effective. We will report an economic analysis including other factors such as consultation time in a separate paper.

Finally, children in the restricted CRP group were younger than those in the CRP for all group. As the clinical prediction rule selects children at higher risk of a serious infection and prevalence of serious infections is higher in younger children, it was to be expected that this would introduce a difference in age between the two groups. Clinician concern was similar between both groups, as were all other baseline variables. (Table 1 of the original paper) [1].
